# Role and mechanism of FOXG1-related epigenetic modifications in cisplatin-induced hair cell damage

**DOI:** 10.3389/fnmol.2023.1064579

**Published:** 2023-04-26

**Authors:** Yu-rong Mu, Sheng-yu Zou, Ming Li, Yan-yan Ding, Xiang Huang, Zu-hong He, Wei-jia Kong

**Affiliations:** ^1^Department of Otorhinolaryngology, Union Hospital, Tongji Medical College, Huazhong University of Science and Technology, Wuhan, China; ^2^Department of Otorhinolaryngology-Head and Neck Surgery, Zhongnan Hospital of Wuhan University, Wuhan, China

**Keywords:** cisplatin, ototoxicity, hair cells, FOXG1, epigenetics, autophagy

## Abstract

Cisplatin is widely used in clinical tumor chemotherapy but has severe ototoxic side effects, including tinnitus and hearing damage. This study aimed to determine the molecular mechanism underlying cisplatin-induced ototoxicity. In this study, we used CBA/CaJ mice to establish an ototoxicity model of cisplatin-induced hair cell loss, and our results showed that cisplatin treatment could reduce FOXG1 expression and autophagy levels. Additionally, H3K9me2 levels increased in cochlear hair cells after cisplatin administration. Reduced FOXG1 expression caused decreased microRNA (miRNA) expression and autophagy levels, leading to reactive oxygen species (ROS) accumulation and cochlear hair cell death. Inhibiting miRNA expression decreased the autophagy levels of OC-1 cells and significantly increased cellular ROS levels and the apoptosis ratio *in vitro*. *In vitro*, overexpression of FOXG1 and its target miRNAs could rescue the cisplatin-induced decrease in autophagy, thereby reducing apoptosis. BIX01294 is an inhibitor of G9a, the enzyme in charge of H3K9me2, and can reduce hair cell damage and rescue the hearing loss caused by cisplatin *in vivo*. This study demonstrates that FOXG1-related epigenetics plays a role in cisplatin-induced ototoxicity through the autophagy pathway, providing new ideas and intervention targets for treating ototoxicity.

## Introduction

Cisplatin is widely used to treat tumors but frequently causes ototoxicity, including tinnitus and hearing loss (Lanvers-Kaminsky et al., 2017). Cisplatin-related ototoxicity is cumulative with dose and time (Keilty et al., 2021) and may be related to various factors, such as DNA damage, oxidative stress, and cellular inflammatory factors (Wu et al., 2021). Cisplatin is widely used in clinical practice despite the risk of ototoxicity (Kros and Steyger, 2019). While numerous studies have been conducted, the mechanism underlying cisplatin-induced ototoxicity remains elusive.

Forkhead box G1 (FOXG1) plays an important role in the development of hair cells (HCs) and supporting cells and the innervation of cochlear and vestibular neuron (Pauley et al., 2006; Zhang et al., 2020). However, the exact role of FOXG1 in cisplatin-induced ototoxic injury remains unclear. In this study, we use a cisplatin-induced HC damage model to determine the underlying mechanism of FOXG1 in ototoxicity.

Autophagy is an essential intracellular process that transports cytoplasmic substances to lysosomes for degradation (Klionsky et al., 2021). Previous studies have shown that FOXG1 plays an important role in the process of hearing degradation by regulating autophagy (He et al., 2021). BIX01294 is an inhibitor of G9a, the enzyme in charge of Histone H3 lysine 9 dimethylation (H3K9me2), and can induce autophagy in various cell types, including neuroblastoma (Ke et al., 2014), glioma stem-like (Ciechomska et al., 2016), oral squamous cell carcinoma (Ren et al., 2015), breast and colon cancer (Kim et al., 2013), and osteosarcoma (Fan et al., 2015) cells. Many microRNAs (miRNAs) regulate the autophagy pathway and influence body processes (Mao et al., 2018; Yuan et al., 2018; Chen et al., 2019; Xie et al., 2019; Khodakarimi et al., 2021). In addition, BIX01294 can reduce HCs loss in organ of Corti explant under cisplatin treatment (Yu et al., 2013). The roles of FOXG1, autophagy, and H3K9me2 in mammalian hair cells and their interrelationships in cisplatin ototoxicity require further exploration.

H3K9me2 modifications and miRNA activities are epigenetic processes. Epigenetics is the regulation of gene expression programs in conjunction with DNA templates including DNA modification, histone modification, and noncoding RNA regulation (Han and He, 2016). H3K9me2 levels increase after neomycin and cisplatin is applied to the cochlea; however, this increase disappears after prolonged neomycin action, indicating that changes in H3K9me2 levels after HC injuries are dynamic (Yu et al., 2013). Epigenetic modifications play an important role in development, protection, and regeneration of inner ear (Layman and Zuo, 2014).

In the present study, we analyzed the roles and mechanisms of FoxG1 and epigenetics in cisplatin-induced hair cell loss in CBA/CaJ mouse model. Our data show that FOXG1 regulates autophagy levels under cisplatin-induced HC injury and that FOXG1 overexpression activates autophagy after cisplatin treatment. We also evaluated H3K9me2 levels in OC-1 cells and cochlea after cisplatin treatment and found that H3K9me2 affects autophagy through FOXG1, affecting the ability of autophagy-related miRNAs to regulate autophagy. Inhibition of H3K9me2 helps reduce hearing and hair cell loss induced by cisplatin *in vivo*. This study demonstrates the important roles of FOXG1 and epigenetics in cisplatin-induced ototoxicity through the autophagy pathway, providing a new target for investigating cisplatin-associated ototoxicity.

## Results

### Construction of a cisplatin-induced ototoxicity animal model

**Figure 1:**
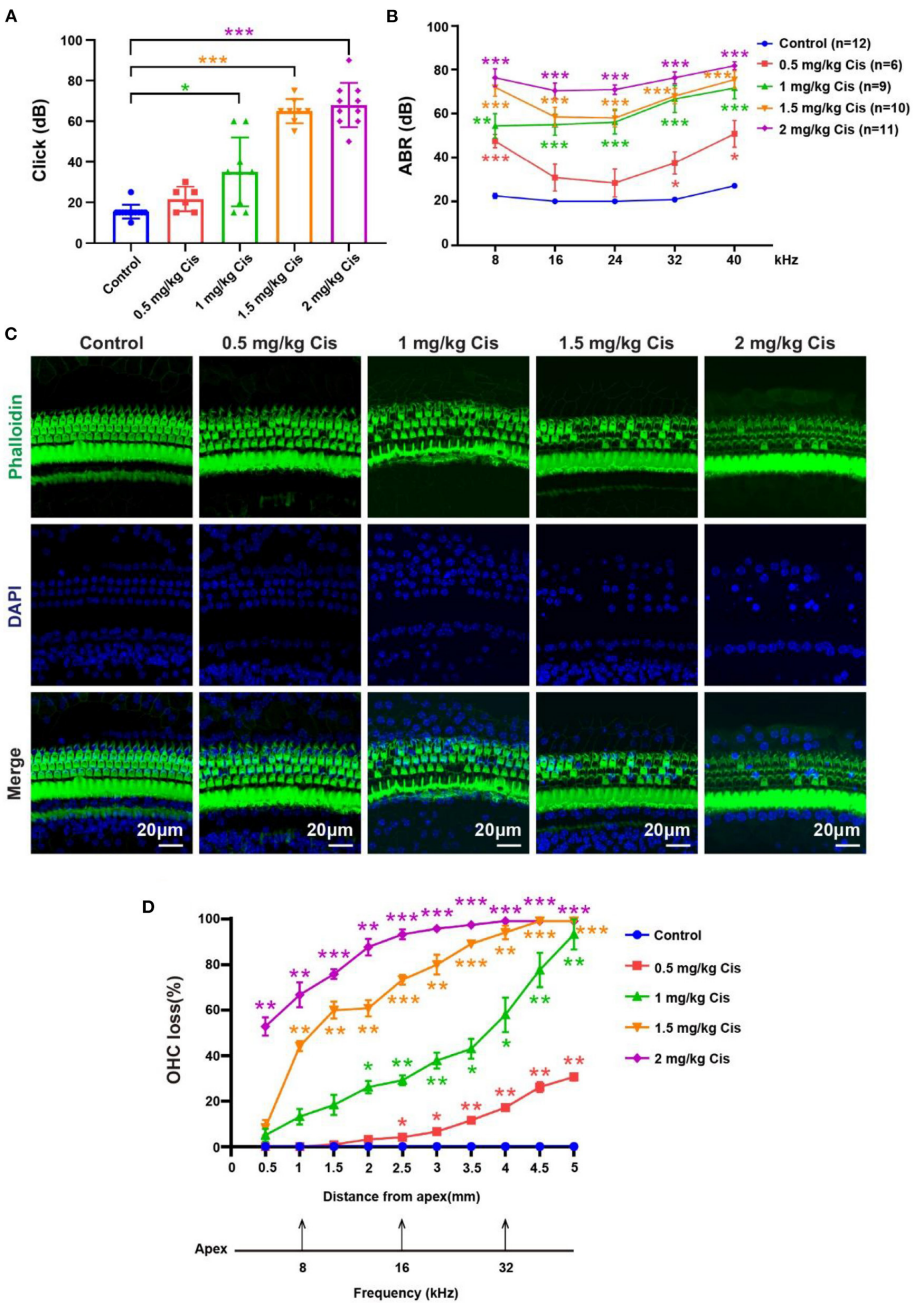
Results of the hearing loss model treated with different cisplatin concentrations. **(A)** Statistical scatter plot of the click ABR. **(B)** Statistical line chart of the tone burst ABR. **(C)** Immunofluorescence staining with phalloidin and DAPI in the cochlea. **(D)** Quantification of the immunofluorescence staining in the cochlea. *p < 0.05, **p < 0.01, ***p < 0.001.

Furosemide transiently decreases the red blood cell count in the stria vascularis on the cochlear lateral wall, allowing cisplatin to easily pass through the blood–ear barrier into the cochlea (Li et al., 2011). We administered furosemide at 200 mg/kg (intraperitoneal) and cisplatin at different concentrations (0.5, 1, 1.5, and 2 mg/kg, subcutaneous) daily for three consecutive days to CBA/CaJ mice to create the model, followed by 3 days of post-treatment recovery. Mouse hearing was evaluated based on the auditory brainstem response (ABR). The threshold of click ABR in the treatment groups (mice treated with different cisplatin concentrations) was higher than that in the control group to different degrees (Figure 1A, *p* < 0.05). In the treatment groups, the tone burst ABR showed varying degrees of loss at 8, 16, 24, 32, and 40 kHz, and the tone burst ABR loss increased as cisplatin concentrations increased (Figure 1B, *p* < 0.05). After modeling, the cochlea was dissected, and cochlear HC loss was assessed via immunofluorescence staining with phalloidin and DAPI. Outer HCs loss in the cochlea worsened with increasing cisplatin concentrations (Figures 1C, D, *p* < 0.05, *n* = 3). The loss of outer HCs began from the base turn and spread toward the apex of the sensory epithelium of Corti as the cisplatin concentration increased.

### OC-1 cell viability decreases with increasing cisplatin concentrations and treatment times

**Figure 2:**
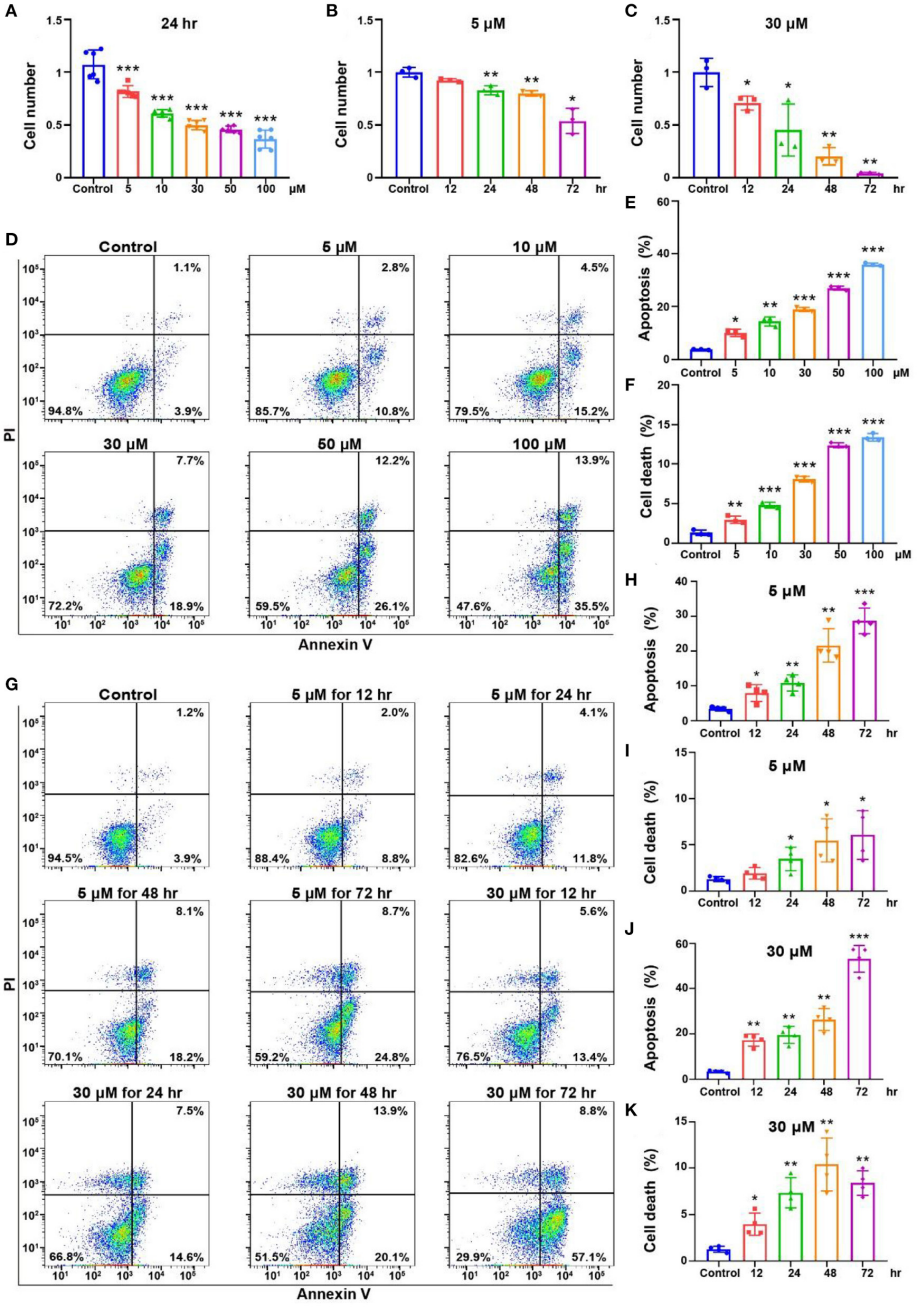
Cell viability and apoptosis flow cytometry results for the OC-1 cells treated with different concentrations of cisplatin. **(A)** CCK-8 cell viability changes after different concentrations of cisplatin treatment for 24 h. **(B)** CCK-8 cell viability changes after 5 μM cisplatin treatment for different times. **(C)** CCK-8 cell viability changes after 30 μM cisplatin treatment for different times. **(D)** Flow cytometry of apoptosis in cells treated with different cisplatin concentrations for 24 h. **(E)** Quantification of the apoptosis ratio in **(D)**. **(F)** Quantification of the cell death ratio in **(D)**. **(G)** Flow cytometry of cells treated with 5 or 30 μM cisplatin for different times. **(H)** Quantification of the apoptosis ratio in **(G)**. **(I)** Quantification of the cell death in **(G)**. **(J)** Quantification of the apoptosis ratio in **(G)**. **(K)** Quantification of the cell death ratio in **(G)**. *p < 0.05, **p < 0.01, ***p < 0.001.

HEI-OC1 is one of the most commonly used mouse auditory cell lines suitable for exploring ototoxic drug models (Kalinec et al., 2016). We treated OC-1 cells with cisplatin at different concentrations and times to construct the cisplatin-induced OC-1 cell cytotoxicity model. First, we treated OC-1 cells with 5, 10, 30, 50, and 100 μM cisplatin for 24 h and detected their viability with CCK-8. Viable OC-1 cell numbers gradually decreased with increasing cisplatin concentrations. Approximately 50% of the OC-1 cells were viable 24 h after 30 μM cisplatin treatment (Figure 2A, *p* < 0.001, *n* = 6).

Then, we treated OC-1 cells with 5 μM cisplatin for 12, 24, 48, and 72 h and assessed cell viability with CCK-8. Viable OC-1 cell numbers gradually decreased as the treatment time increased, significantly decreasing at 72 h (Figure 2B, *p* < 0.05, *n* = 3). Subsequently, we treated OC-1 cells with 30 μM cisplatin for 12, 24, 48, and 72 h and assessed cell viability with CCK-8. Viable OC-1 cell numbers decreased significantly over time with this higher cisplatin concentration (Figure 2C, *p* < 0.05, *n* = 3).

Next, we treated OC-1 cells with 5, 10, 30, 50, and 100 μM cisplatin for 24 h and labeled apoptotic cells with annexin V and dead cells with propidium iodide (PI) to assess the apoptotic and dead cell ratio by flow cytometry. The apoptotic and dead cell ratios of OC-1 cells gradually increased as the cisplatin concentration increased (Figures 2D–F, *p* < 0.01, *n* = 3). Additionally, OC-1 cells were treated with low (5 μM) or high (30 μM) cisplatin concentrations for 12, 24, 48, and 72 h before flow cytometry. The apoptotic and dead cell ratios of OC-1 cells gradually increased as the cisplatin treatment time increased (Figures 2G–K, *p* < 0.05, *n* = 3).

**Figure 3:**
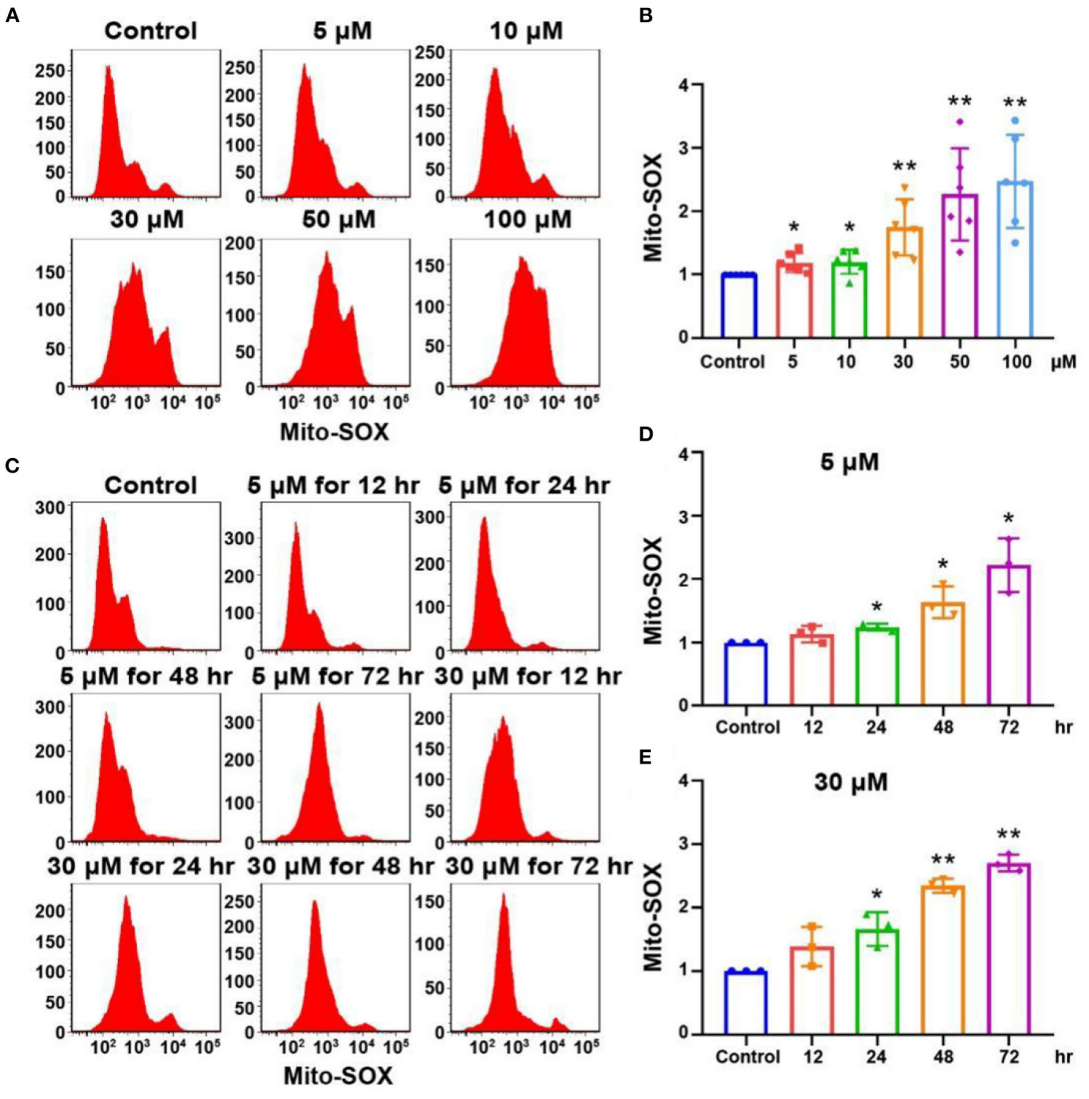
Mito-SOX flow cytometry of OC-1 cells treated with different cisplatin concentrations and times. **(A)** ROS levels in cells treated with different cisplatin concentrations for 24 h. **(B)** Quantification of the data in A. **(C)** ROS levels in cells treated with 5 or 30 μM cisplatin for different times. **(D)** Quantification of the data in **(C)**. **(E)** Quantification of the data in **(C)**. *p < 0.05, **p < 0.01.

The accumulation of mitochondrial superoxide in cells can induce DNA damage and ultimately lead to cell damage (Srinivas et al., 2019). We detected the level of oxidative stress in OC-1 cells by Mito-SOX flow cytometry. After treatment with 5, 10, 30, 50, and 100 μM cisplatin for 24 h, the flow cytometry results showed that ROS levels in OC-1 cells gradually increased as the cisplatin concentration increased (Figures 3A, B, *p* < 0.01, *n* = 3). Next, OC-1 cells were treated with low (5 μM) or high (30 μM) cisplatin concentrations for 12, 24, 48, and 72 h before Mito-SOX flow cytometry. The low cisplatin concentration (5 μM) slightly increased ROS levels in OC-1 cells over time, becoming significant after a longer period (Figures 3C, D, *p* < 0.05, *n* = 3). The high cisplatin concentration (30 μM) significantly increased ROS levels in OC-1 cells over time (Figures 3C, E, *p* < 0.05, *n* = 3). After cisplatin treatment, the level of ROS in OC-1 cells increased, indicating that ROS accumulates in OC-1 cells as cisplatin concentrations and treatment times increase.

### FOXG1 expression and autophagy level are altered by cisplatin treatment

FOXG1 is a nuclear transcription factor that participates in morphogenesis, cell fate determination, and proliferation and is required for mammalian inner ear morphogenesis (Zhang et al., 2020). FOXG1 is related to the survival of HCs; however, the specific downstream pathways and mechanisms are unclear. FOXG1 is related to mitochondrial function and metabolism, as is autophagy (He et al., 2020). Autophagy is an essential intracellular process that transports cytoplasmic substances to lysosomes for degradation (Klionsky et al., 2021). It plays a crucial role in adaptive responses to starvation and other forms of stress (Jiang and Mizushima, 2014). Autophagy is involved in multiple signaling pathways and contributes to HC development and protection. With autophagy pathway activation, autophagosomes can envelop the damaged mitochondria and fuse with lysosomes to form autolysosomes, degrading the damaged mitochondria and promoting HC survival (He et al., 2017).

**Figure 4:**
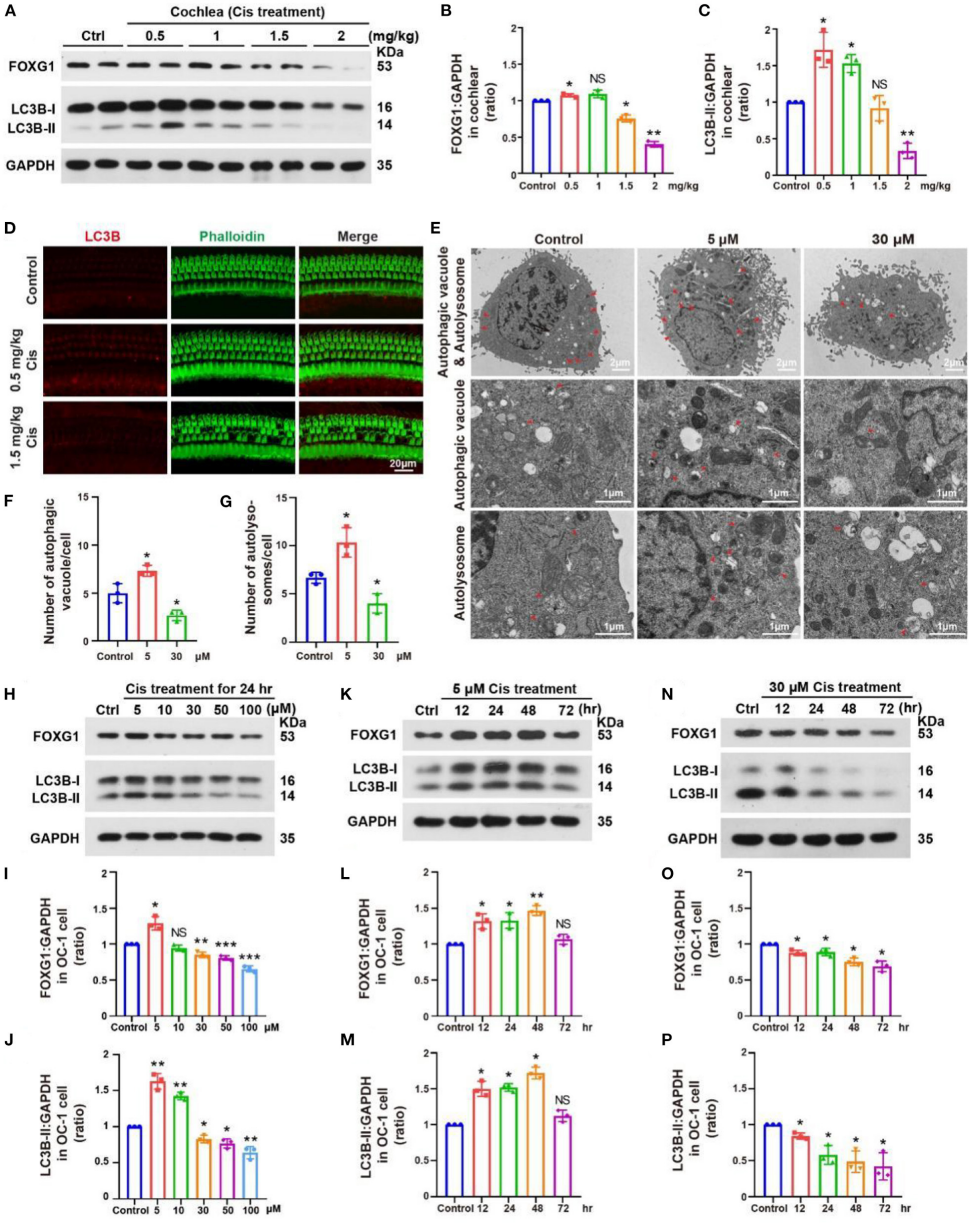
Changes in FOXG1 and autophagy levels in the cochlea and OC-1 cells after cisplatin treatment. **(A)** Western blotting of changes in FOXG1 and LC3B levels in the cochlea after treatment with different cisplatin concentrations. **(B, C)** Quantification of the western blotting in **(A)**. **(D)** Immunofluorescence staining for LC3B and phalloidin in the cochlea after cisplatin treatment. **(E)** TEM of changes in autophagic vacuoles and autolysosomes after treatment with 5 or 30 μM cisplatin for 24 h. **(F)** Quantification of the autophagic vacuoles changes in **(E)**. **(G)** Quantification of the autolysosomes changes in **(E)**. **(H)** Western blotting of changes in FOXG1 and LC3B levels after treatment with different cisplatin concentrations for 24 h. **(I, J)** Quantification of the western blotting in **(H)**. **(K)** Western blotting of changes in FOXG1 and LC3B levels after treatment with 5 μM cisplatin for different times. **(L, M)** Quantification of the western blotting in **(K)**. **(N)** Western blotting of changes in FOXG1 and LC3B levels after treatment with 30 μM cisplatin for different times. **(O, P)** Quantification of the western blotting in **(N)**. *p < 0.05, **p < 0.01, ***p < 0.001.

To investigate changes in FOXG1 and autophagy levels after cisplatin treatment in a mouse model, we administered furosemide and different cisplatin concentrations. We dissected the cochlea 3 days post-cisplatin treatment and extracted proteins for western blotting. FOXG1 and LC3B levels in the cochlea increased relative to the control after treatment with low cisplatin concentrations but decreased with high cisplatin concentrations (Figures 4A–C, *p* < 0.05, *n* = 3). Immunofluorescence staining showed similar LC3B levels in the cochlea after cisplatin treatment to those observed by western blotting. LC3B fluorescence intensity increased after treatment with 0.5 mg/kg cisplatin but decreased with 1.5 mg/kg cisplatin (Figure 4D, *p* < 0.05, *n* = 3).

We next treated in OC-1 cells with 5 and 30 μM cisplatin for 24 h and performed transmission electron microscopy (TEM) to confirm the changes in autophagy after cisplatin treatment. The 5 μM cisplatin group demonstrated a significantly higher number of autophagic vacuoles and autolysosomes than the control group. In contrast, the 30 μM cisplatin treatment group showed a significantly lower number of autophagic vacuoles and autolysosomes than the control group (Figures 4E–G, *p* < 0.05, *n* = 3).

Next, we treated OC-1 cells with 5, 10, 30, 50, and 100 μM cisplatin for 24 h and detected changes in FOXG1 and LC3B levels. Western blotting showed that FOXG1 levels increased with 5 μM cisplatin relative to the control but decreased when the cisplatin concentrations exceeded 30 μM. Similarly, LC3B levels increased with 5 and 10 μM cisplatin treatment before gradually decreasing when the cisplatin concentrations exceeded 30 μM. These findings indicate that autophagy levels initially increase and then decrease as cisplatin concentration increases (Figures 4H–J, *p* < 0.05, *n* = 3).

Then, we treated OC-1 cells with 5 μM cisplatin for 12, 24, 48, and 72 h and detected changes in FOXG1 and LC3B levels. Western blotting showed that FOXG1 and LC3B levels were increased relative to the control in the treatment group at 48 h; however, no significant differences were observed between the treatment and control groups at 72 h (Figures 4K–M, *p* < 0.05, *n* = 3). Finally, we repeated this experiment with 30 μM cisplatin. Western blotting showed that FOXG1 and LC3B levels gradually decreased after cisplatin treatment (Figures 4N–P, *p* < 0.05, *n* = 3). These results suggest that FOXG1 plays an important protective role against cisplatin-induced ototoxic damage in OC-1 cells. However, FOXG1 and autophagy levels are significantly reduced after the cell damage exceeds its repair capacity.

We found that cisplatin treatment could affect FOXG1 expression and autophagy pathway. Our previous study showed that FOXG1 could regulate the autophagy pathway in presbycusis (He et al., 2021). Here, low cisplatin doses activated FOXG1 expression and the autophagy pathway. As the cisplatin concentration gradually increased, FOXG1 and LC3B expression levels decreased. Therefore, we speculate that low concentrations of cisplatin activate the cells' self-defense mechanism, increasing FOXG1 expression and activating the autophagy pathway to eliminate ROS in OC-1 cells. With high concentrations of cisplatin, cells gradually lose their self-defense ability, significantly reducing FOXG1 expression and autophagy levels.

### H3K9me2 changes in HCs after cisplatin treatment

Epigenetic modifications have recently been found to contribute to inner ear development and HC regeneration (Taiber et al., 2022). Histone methylation and demethylation are implicated in transcriptional regulation, genome integrity, and epigenetics (Klose and Zhang, 2007). H3K9 methylation is critical for early embryogenesis and is involved in the transcriptional repression of developmental genes (Tachibana et al., 2002).

**Figure 5:**
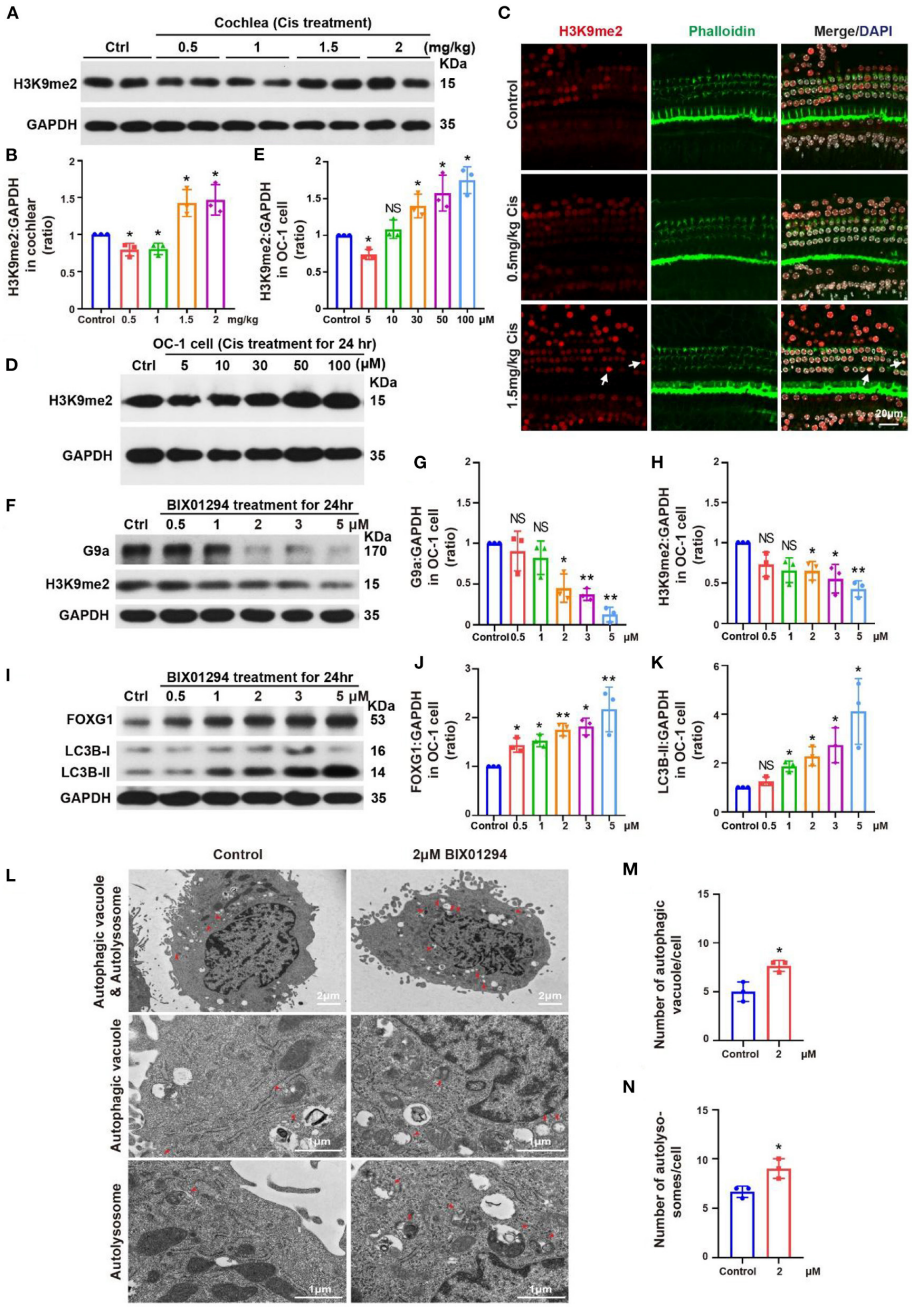
Changes in H3K9me2 levels in the cochlea and OC-1 cells after treatment with different cisplatin concentrations. **(A)** Western blotting of changes in H3K9me2 levels in the cochlea after treatment with different cisplatin concentrations. **(B)** Quantification of the western blotting in **(A)**. **(C)** Immunofluorescence staining for H3K9me2, phalloidin, and DAPI in the cochlea after cisplatin treatment. **(D)** Western blotting of changes in H3K9me2 levels after treatment with different cisplatin concentrations for 24 h. **(E)** Quantification of the western blotting in **(D)**. **(F)** Western blotting of changes in H3K9me2 levels with different BIX01294 concentrations for 24 h. **(G, H)** Quantification of the western blotting in **(F)**. **(I)** Western blotting of changes in FOXG1 and LC3B levels after treatment with different BIX01294 concentrations for 24 h. **(J, K)** Quantification of the western blotting in **(I)**. **(L)** TEM of the changes in autophagic vacuoles and autolysosomes after treatment with 2 μM BIX01294 for 24 h. **(M)** Quantification of the autophagic vacuoles changes in **(L)**. **(N)** Quantification of the autolysosomes changes in **(L)**. *p < 0.05, **p < 0.01.

We treated the mouse model with different cisplatin concentrations. At 3 days post-cisplatin treatment, we detected changes in H3K9me2 levels in the cochlea, which were decreased relative to the control at low cisplatin concentrations (0.5 and 1 mg/kg) but increased at high cisplatin concentrations (1.5 and 2 mg/kg; Figures 5A, B, *p* < 0.05, *n* = 3). Immunofluorescence staining showed similar H3K9me2 levels in the cochlea after cisplatin treatment to those observed by western blotting. H3K9me2 fluorescence intensity was decreased with 0.5 mg/kg cisplatin but increased with 1.5 mg/kg cisplatin (Figure 5C, *p* < 0.05, *n* = 3). We then treated OC-1 cells with 5, 10, 30, 50, and 100 μM cisplatin for 24 h and detected H3K9me2 levels. H3K9me2 levels decreased with 0.5 μM cisplatin relative to control but increased when cisplatin concentrations exceeded 10 μM (Figures 5D, E, *p* < 0.05, *n* = 3). H3K9me2 levels decreased relative to control after low-concentration cisplatin treatment *in vivo* and *in vitro*. When the concentration of cisplatin increased, the level of H3K9me2 increased, and the expression of FOXG1 and the autophagy pathway were inhibited.

BIX01294 is an inhibitor of euchromatic histone methyltransferase G9a that can transiently and reversibly inhibit H3K9me2 activity by competing with G9a for substrates (Kubicek et al., 2007; Kondengaden et al., 2016; Milite et al., 2019). H3K9me2 inhibition by BIX01294 can induce autophagy in various cell types, including glioblastoma cells (Ciechomska et al., 2016). Previous studies have shown that BIX01294 can reduce HC loss in organ of Corti explant under cisplatin treatment (Yu et al., 2013).

We treated OC-1 cells with different concentrations (0.5, 1, 2, 3, and 5 μM) of the G9a inhibitor BIX01294 for 24 h to inhibit H3K9me2 levels and detected their viability with CCK-8. Viable OC-1 cell numbers gradually decreased as the BIX01294 concentration increased (Supplementary Figure 1A, *p* < 0.05, *n* = 6). We performed flow cytometry on the BIX01294-treated OC-1 cells to detect changes in the ratios of apoptotic and dead cells. The apoptotic and dead cell ratios of OC-1 cells increased significantly as BIX01294 concentrations increased (Supplementary Figures 1B–D, *p* < 0.05, *n* = 3). We also performed Mito-SOX flow cytometry to detect mitochondrial ROS in the OC-1 cells treated with BIX01294. ROS levels in OC-1 cells increased significantly as BIX01294 concentrations increased (Supplementary Figures 2A, B, *p* < 0.05, *n* = 3).

Next, we treated OC-1 cells with different BIX01294 concentrations (0.5, 1, 2, 3, and 5 μM) for 24 h and detected G9a and H3K9me2 levels. G9a and H3K9me2 levels gradually decreased as BIX01294 concentrations increased (Figures 5F–H, *p* < 0.05, *n* = 3). We then detected FOXG1 and LC3B levels, which gradually increased as BIX01294 concentrations increased (Figures 5I–K, *p* < 0.05, *n* = 3). To confirm the changes in autophagy after BIX01294 treatment, we treated OC-1 cells with 2 μM BIX01294 for 24 h and performed TEM. The 2 μM BIX01294 treatment group showed significantly increased autophagic vacuoles and autolysosomes compared to the control group (Figures 5L–N, *p* < 0.05, *n* = 3). FOXG1 and LC3B levels significantly increased after BIX01294 treatment, indicating that H3K9me2 may be an upstream regulator of FOXG1 and autophagy. After treating OC-1 cells with BIX01294, the H3K9me2 level decreased significantly, and the expressions of FOXG1 and LC3B increased significantly. Overall, FOXG1 and autophagy levels increased and H3K9me2 levels decreased in response to low-concentration cisplatin. At high concentrations of cisplatin, the levels of FOXG1 and autophagy decreased, while the level of H3K9me2 increased. These findings suggest that H3K9me2 may be the upstream regulator of FOXG1 and autophagy.

### H3K9me2 may regulate autophagy pathway activation through FOXG1

**Figure 6:**
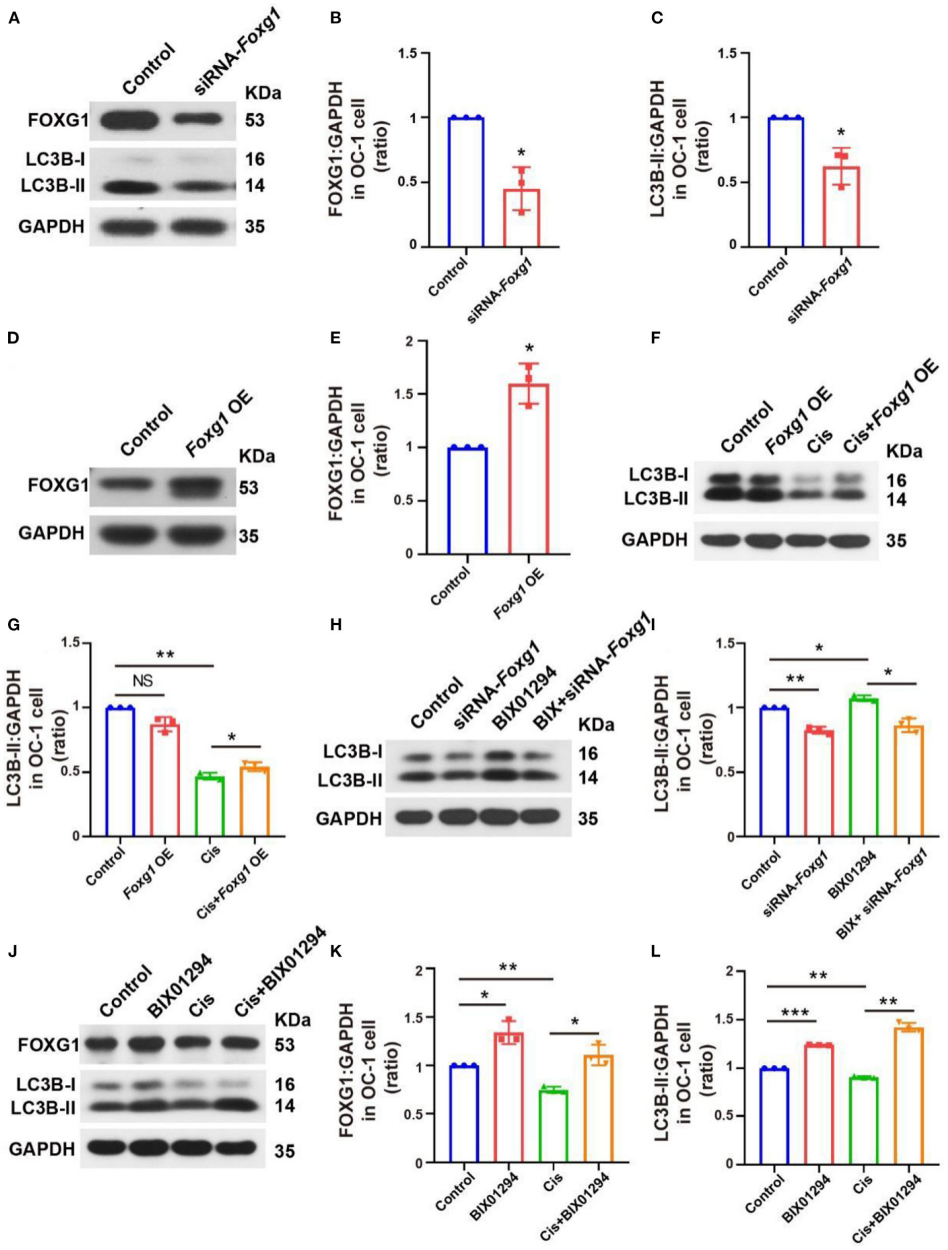
FOXG1 and BIX01294 may regulate autophagy pathway activation, and the BIX01294-induced autophagy activation pathway may be FoxG1-dependent. **(A)** Western blotting of FOXG1 and LC3B levels after *Foxg1* knock down. **(B, C)** Quantification of the western blotting in **(A)**. **(D)** Western blotting of the *Foxg1* overexpression plasmid's transfection efficiency. **(E)** Quantification of the western blotting in **(D)**. **(F)** Western blotting LC3B levels with *Foxg1* overexpression and cisplatin treatment. **(G)** Quantification of the western blotting in **(F)**. **(H)** Western blotting of LC3B levels with *Foxg1* knockdown and BIX01294 treatment. **(I)** Quantification of the western blotting in **(H)**. **(J)** Western blotting of FOXG1 and LC3B levels after cisplatin and BIX01294 treatment. **(K, L)** Quantification of the western blotting in **(J)**. *p < 0.05, **p < 0.01, ***p < 0.001.

We used small interfering RNAs (siRNAs) to knock down *Foxg1* expression in OC-1 cells and quantified FOXG1 and LC3B levels. LC3B levels decreased significantly after FOXG1 knockdown (Figures 6A–C, *p* < 0.05, *n* = 3). Next, we constructed a plasmid to overexpress FOXG1 in OC-1 cells and found that FOXG1 expression increased after plasmid transfection (Figures 6D, E, *p* < 0.05, *n* = 3). Then, we treated OC-1 cells transfected with the *Foxg1*-overpressing plasmid with cisplatin; this overexpression rescued the decrease in autophagy levels caused by cisplatin (Figures 6F, G, *p* < 0.05, *n* = 3). These results indicate that FOXG1 might upregulate autophagy during cisplatin injury.

We further explored whether FOXG1 silencing inhibited BIX01294-induced LC3B activation and found that FOXG1 was required for BIX01294-induced autophagy activation (Figures 6H, I, *p* < 0.05, *n* = 3). Finally, we treated OC-1 cells with BIX01294 followed by cisplatin and found that BIX01294 prevented the decreases in FOXG1 and autophagy levels caused by cisplatin (Figures 6J–L, *p* < 0.05, *n* = 3). These results indicate that H3K9me2 may regulateg autophagy through a FOXG1-dependent pathway.

### FOXG1 and H3K9me2 regulate autophagy pathway activation through autophagy-related miRNAs

Many microRNAs (miRNAs) regulate the autophagy pathway and influence various body processes (Khodakarimi et al., 2021), including miR-34a, miR-96, miR-182, and miR-183, which are associated with apoptosis and autophagy. For example, miR-34 overexpression significantly decreased ovarian cancer cell proliferation by activating apoptosis and autophagy (Jia et al., 2019). miR-96 overexpression inhibits autophagosome formation in the hippocampus by inhibiting Atg7 and Atg16L1 expression (Gan et al., 2017). Reduced miR-182 expression inhibits RAB10 expression, reducing cell viability and autophagy and promoting apoptosis in gastric cancer cells (Duan et al., 2022). miR-183 knockdown in medullary thyroid carcinoma increases LC3B expression, reducing cell proliferation (Abraham et al., 2011). Therefore, we studied the relationships among FOXG1, H3K9me2, and autophagy-related miRNAs.

**Figure 7:**
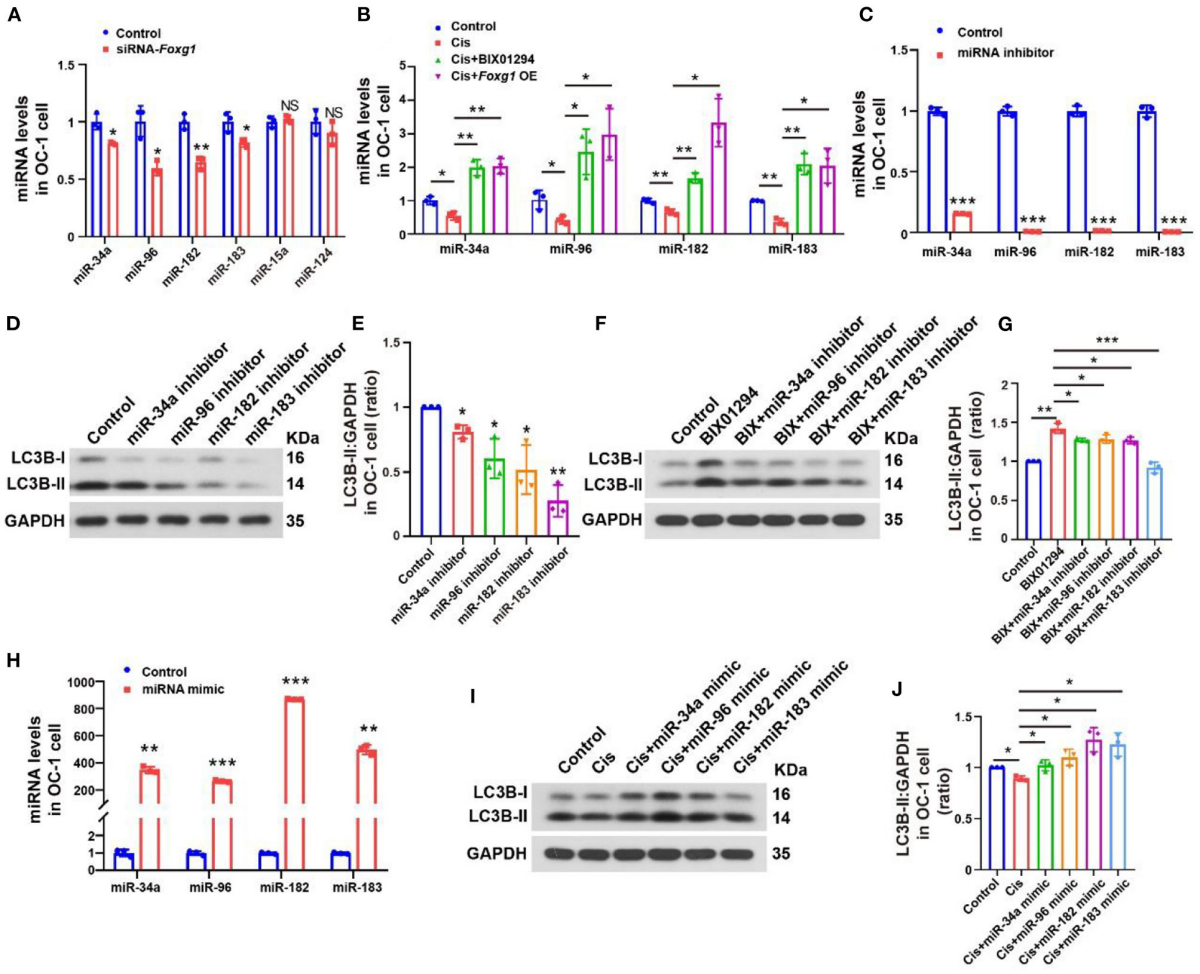
Autophagy levels of OC-1 cells change through the regulation of miR-34a, miR-96, miR-182, and miR-183 expression. **(A)** rt-PCR of changes in miR-34a, miR-96, miR-182, and miR-183 expression with *Foxg1* knockdown **(B)** rt-PCR of the expression in miR-34a, miR-96, miR-182, and miR-183 changes after cisplatin treatment with BIX01294 pre-treatment or *Foxg1* overexpression. **(C)** rt-PCR of miR-34a, miR-96, miR-182, and miR-183 expression inhibition efficiency. **(D)** Western blotting of changes in LC3B levels after miR-34a, miR-96, miR-182, and miR-183 inhibition. **(E)** Quantification of the western blotting in **(F)**. **(F)** Western blotting of changes in LC3B levels after BIX01294 treatment with miR-34a, miR-96, miR-182, and miR-183 inhibition. **(G)** Quantification of the western blotting in **(F)**. **(H)** rt-PCR of miRNA mimic efficiency for enhancing miR-34a, miR-96, miR-182, and miR-183 expression. **(I)** Western blotting of changes in LC3B levels after cisplatin treatment with miR-34a, miR-96, miR-182, and miR-183 mimics. **(J)** Quantification of the western blotting in **(F)**. *p < 0.05, **p < 0.01, ***p < 0.001.

We extracted RNA from OC-1 cells after siRNA-*Foxg1* treatment and detected changes in several autophagy-related miRNAs with rt-PCR. miR-34a, miR-96, miR-182, and miR-183 expression levels decreased FOXG1 expression decreased. In contrast, miR-15a and miR-124 levels did not change significantly (Figure 7A, *p* < 0.05, *n* = 3). We treated OC-1 cells pretreated with BIX01294 or overexpressing FOXG1 with 30 μM cisplatin for 24 h and extracted RNA to detect miR-34a, miR-96, miR-182, and miR-183 expression levels. miR-34a, miR-96, miR-182, and miR-183 levels decreased after cisplatin treatment, and pre-treatment with BIX01294 or FOXG1 overexpression prevented this decrease (Figure 7B, *p* < 0.05, *n* = 3).

Next, we studied the regulatory effect of miRNAs on the autophagy pathway. We used an miRNA inhibitor to inhibit target miRNA expression in OC-1 cells and determined the inhibition efficiency of miR-34a, miR-96, miR-182, and miR-183 by rt-PCR, revealing significantly inhibited expression (Figure 7C, *p* < 0.001, *n* = 3). Then, we quantified LC3B levels with miR-34a, miR-96, miR-182, and miR-183 inhibition, revealing significantly decreased expression (Figures 7D, E, *p* < 0.05, *n* = 3). Furthermore, we examined BIX01294's regulatory effect on autophagy with miR-34a, miR-96, miR-182, and miR-183 inhibition. LC3B levels were decreased after BIX01294 treatment when these four miRNAs were inhibited (Figures 7F, G, *p* < 0.05, *n* = 3).

miRNA mimics are commonly used to overexpress target miRNAs. We used miRNA mimics to overexpress miR-34a, miR-96, miR-182, and miR-183 in OC-1 cells and assessed their overexpression efficiency with rt-PCR, revealing that they were all significantly overexpressed (Figure 7H, *p* < 0.001). Next, we quantified LC3B levels with miR-34a, miR-96, miR-182, and miR-183 overexpression after cisplatin treatment and found that they were increased (Figures 7I, J, *p* < 0.05, *n* = 3), indicating that overexpressing these miRNAs facilitates autophagy activation under cisplatin treatment.

FOXG1 knockdown decreased the levels of miR-34a, miR-96, miR-182, and miR-183 and inhibited the autophagy pathway. Our results showed that the level of autophagy significantly decreased when the expression of the above miRNAs was inhibited. The expression levels of these miRNAs were inhibited after high-dose cisplatin treatment, and this inhibition could be recovered by BIX01294 treatment or overexpressing FOXG1. BIX01294 could not activate the autophagy pathway when these miRNAs were inhibited. However, the overexpression of the above miRNAs could restore autophagy under cisplatin treatment. Our results showed that miR-34a, miR-96, miR-182, and miR-183 were related to the activation of the autophagy pathway, and FOXG1 autophagy regulation is miRNA-dependent.

### MiRNA levels are associated with apoptosis ratios and ROS levels in OC-1 cells

**Figure 8:**
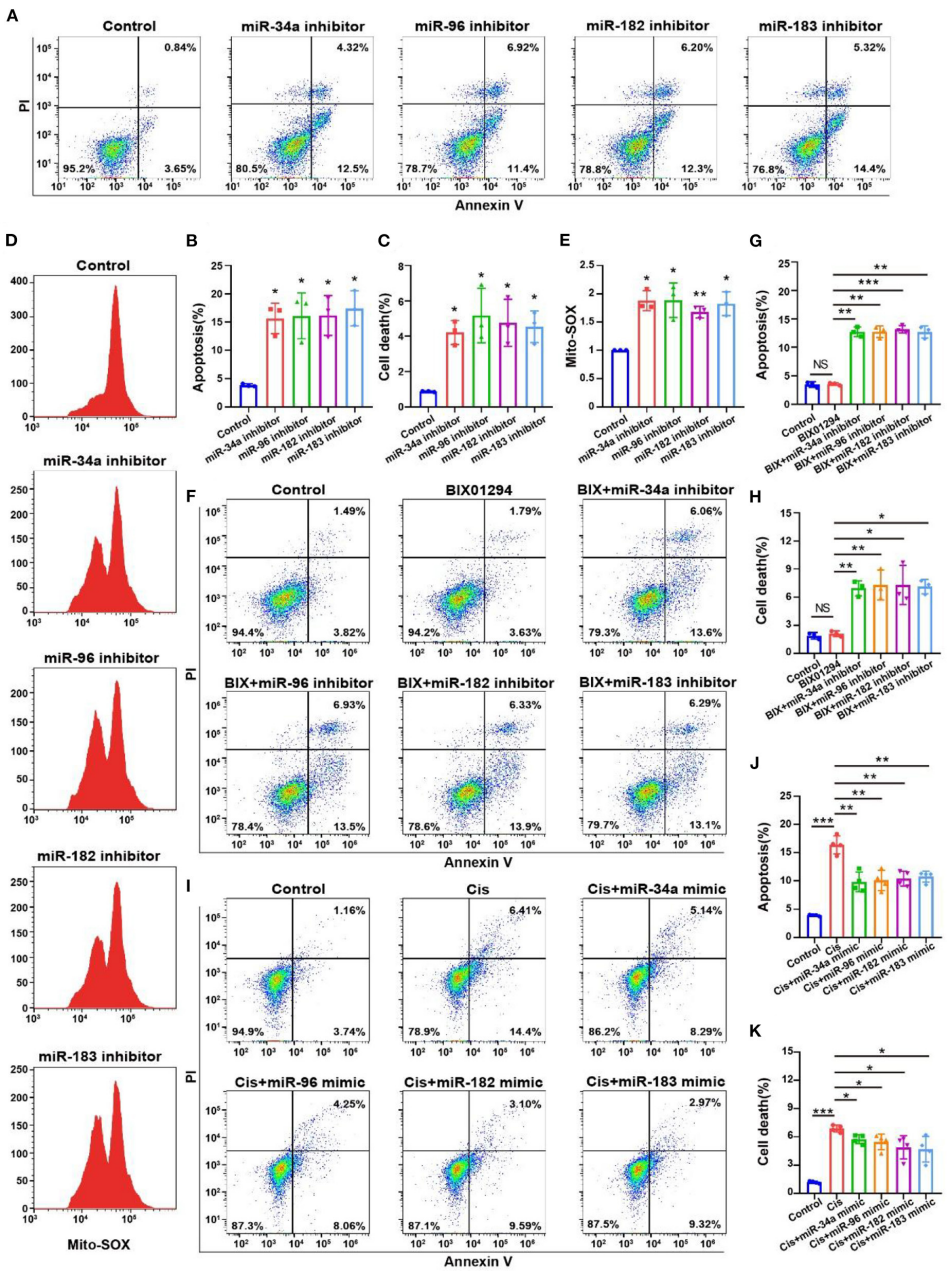
Flow cytometry of apoptosis and ROS levels with miR-34a, miR-96, miR-182, and miR-183 regulation in the OC-1 cells. **(A)** Annexin V flow cytometry of apoptosis with miR-34a, miR-96, miR-182, and miR-183 inhibition. **(B)** Quantification of the apoptosis ratio in **(A)**. **(C)** Quantification of the cell death ratio in **(A)**. **(D)** Mito-SOX flow cytometry of cellular ROS level with miR-34a, miR-96, miR-182, and miR-183 inhibition. **(E)** Quantification of the data in **(D)**. **(F)** Annexin V flow cytometry of apoptosis after BIX01294 treatment with inhibiting miR-34a, miR-96, miR-182, and miR-183. **(G)** Quantification of the apoptosis ratio in **(A)**. **(H)** Quantification of the cell death ratio in **(F)**. **(I)** Annexin V flow cytometry of apoptosis after cisplatin treatment with miR-34a, miR-96, miR-182, and miR-183 mimics. **(J)** Quantification of the apoptosis ratio in **(I)**. **(K)** Quantification of the cell death ratio in **(I)**. *p < 0.05, **p < 0.01, ***p < 0.001.

We performed flow cytometry on OC-1 cells with inhibited miR-34a, miR-96, miR-182, and miR-183 expression to detect changes in the ratios of apoptotic and dead cells and found that these ratios were significantly increased (Figures 8A–C, *p* < 0.05, *n* = 3). We also performed Mito-SOX flow cytometry to detect mitochondrial ROS levels in these cells and found that they were significantly increased (Figures 8D, E, *p* < 0.05, *n* = 3). The apoptosis and death ratios and the ROS levels of the OC-1 cells, significantly increased as the miRNA levels decreased, suggesting that these miRNAs play an important protective role in OC-1 cell survival.

We also found that BIX01294 treatment induced OC-1 apoptosis with miR-34a, miR-96, miR-182 and miR-183 inhibition (Figures 8F–H, *p* < 0.05, *n* = 4). Furthermore, miR-34a, miR-96, miR-182, and miR-183 overexpression reduced cisplatin-induced apoptosis in OC-1 cells, indicating that overexpression of autophagy-related miRNAs protects OC-1 cells from cisplatin-induced injury (Figures 8I–K, *p* < 0.05, *n* = 4). These results indicate that these miRNAs could rescue OC-1 cell damage during cisplatin injury.

### BIX01294 can protect against cisplatin-induced ototoxicity *in vivo*

**Figure 9:**
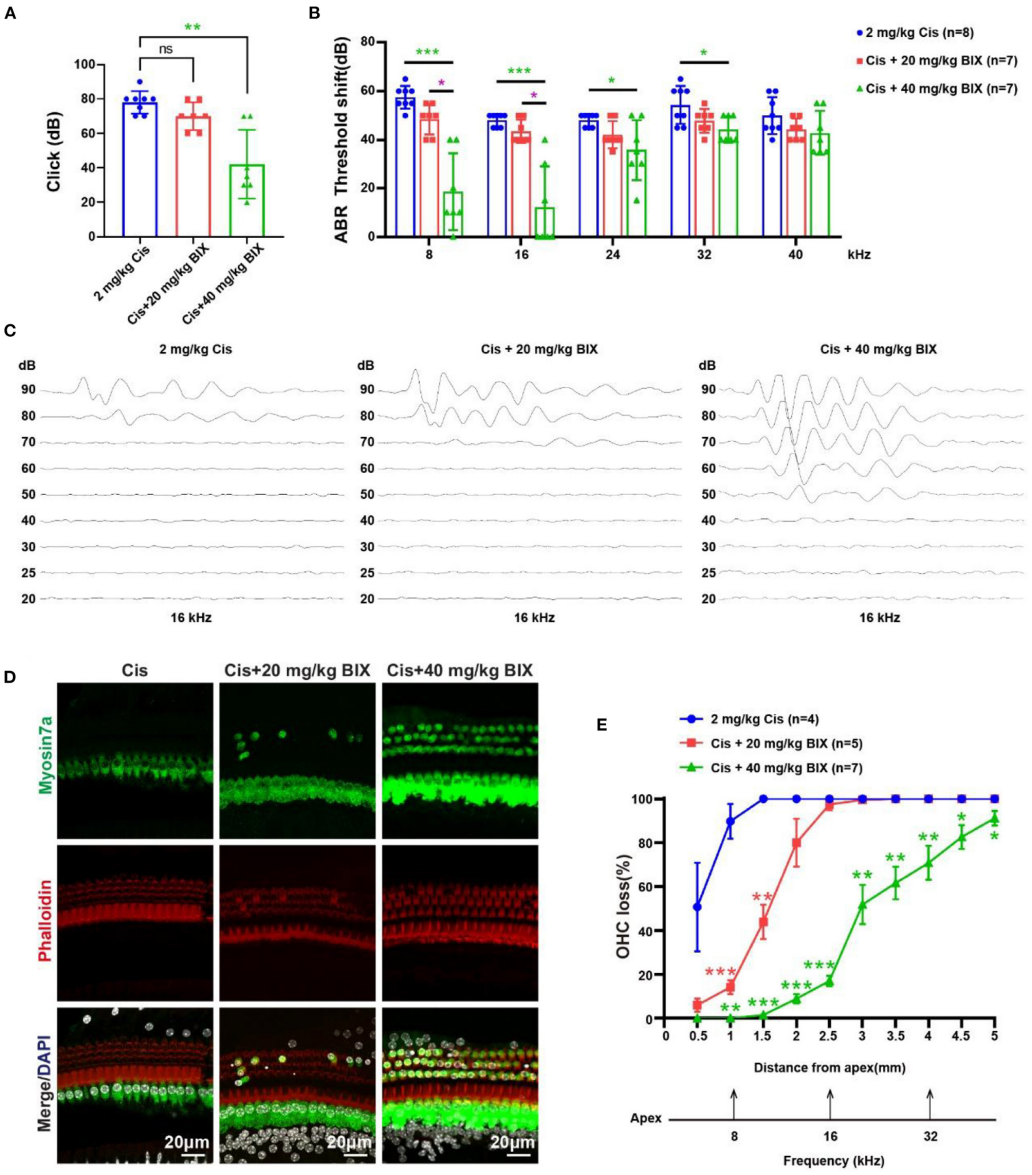
Results of the mouse hearing loss treated with cisplatin and BIX01294. **(A)** Statistical scatter plot of the click ABR. **(B)** Statistical line chart of the tone burst ABR threshold shifts. **(C)** Tone burst ABR at 16 kHz. **(D)** Immunofluorescence staining with myosin7a, phalloidin and DAPI of middle turn in the cochlea. **(E)** Quantification of the immunofluorescence staining in the cochlea. *p < 0.05, **p < 0.01, ***p < 0.001.

To investigate the role of H3K9me2 in hearing during cisplatin-induced injury, we administrated BIX01294 via intraperitoneal injection before and during cisplatin administration *in vivo*. The groups were treated as follows: 2 mg/kg cisplatin, 2 mg/kg cisplatin +20 mg/kg BIX01294, and 2 mg/kg cisplatin +40 mg/kg BIX01294. We administrated BIX01294 via intraperitoneal injection to CBA/CaJ mice on the day before the initiation of cisplatin injections and half an hour before each furosemide injection. The ABR results showed that 40 mg/kg BIX01294 intraperitoneal injection could rescue cisplatin-induced hearing loss, while the hearing changes in the 20 mg/kg BIX01294 group were not significant compared to the cisplatin-only group (Figures 9A, B). The protective effect of BIX01294 on cisplatin-induced hearing loss was more obvious at low-frequencies. BIX01294 rescued the ABR threshold shift at 16 kHz (Figure 9C). We sacrificed the mice after the ABR test, and the cochlea was dissected after fixation and decalcification. We used Myosin 7a and phalloidin to label HCs and quantify HC loss. We observed that the loss of outer HCs in mice in the cisplatin +40 mg/kg BIX01294 group was significantly reduced compared to that in the cisplatin-only group, especially in the apical and middle turns (Figures 9D, E, *p* < 0.05). These results suggest that BIX01294 reduced the ototoxicity caused by cisplatin and protected hearing in CBA/CaJ mice.

## Discussion

Cisplatin is clinically used to treat tumors but has ototoxic side effects. Studying the mechanism of cisplatin-induced ototoxicity is crucial in hearing research. In this study, we conducted related mechanistic experiments since FOXG1-related epigenetics appeared to play a role in cisplatin-induced HC damage.

Auditory system studies have revealed hundreds of miRNAs that are differentially expressed during mammalian inner ear development and aging (Weston et al., 2006; Rudnicki et al., 2014). miRNAs participate in proliferation, apoptosis, and transcription factor regulation, thus playing important roles in organ development and maturation (Harfe, 2005), including sensory organs and systems (e.g., the inner ear and auditory system) (Conte et al., 2013). Transcription factors control miRNA expression at the transcriptional level (Nenna et al., 2022). In an animal acute LPS-induced hearing loss model, the histone deacetylase 2 Hdac2/transcription factor Sp1/miR-204-5p/apoptosis suppressor gene Bcl-2 regulatory axis mediated apoptosis in the cochlea (Xie et al., 2021). FOXG1 is a nuclear transcription factor that participates in morphogenesis and cell fate determination and proliferation and is required for mammalian inner ear morphogenesis (Zhang et al., 2020). In this study, we knocked down FOXG1 expression in OC-1 cells and observed decreased autophagy levels and altered levels of autophagy-related miRNAs, including miR-34, miR-96, miR-182, and miR-183. Autophagy levels also decreased when these miRNAs were inhibited. We demonstrated that reducing FOXG1 expression decreases the autophagy level by reducing miR-34a, miR-96, miR-182, and miR-183 expression levels, leading to cisplatin-induced ototoxicity.

H3K9me2 modification is one of the most abundant and dynamic histone modifications and its levels are highly variable in disease development and pathogenesis (Bhaumik et al., 2007). Studies have shown that BIX01294 can reduce the resistance of tumors to cisplatin by inhibiting H3H9me2 and increase the tumor chemosensitivity by enhancing autophagy (Li et al., 2020; Fu et al., 2023). Herein, we found that cisplatin-induced injury increased H3K9me2 levels, and H3K9me2 inhibition increased FOXG1 expression and autophagy levels in OC-1 cells.

This study demonstrated that inhibition of H3H9me2 by BIX01294 *in vivo* can reduce the damage to the inner ear hair cells caused by cisplatin, indicating that the regulation of epigenetics can reduce the ototoxicity of cisplatin *in vivo*. Therefore, whether overexpression of FOXG1 and miRNA *in vivo* can also reduce the ototoxicity of cisplatin will become a new research goal in ototoxicity prevention and treatment. Many inner ear gene therapy methods exist for cochlear hair cells and supporting cells, such as synthetic adeno-associated virus approaches (Zhu et al., 2019). Because of the low transduction rate of adeno-associated virus in the cochlea, researchers have designed AAV-inner ear for gene delivery in the mouse cochlea and achieved a good therapeutic effect (Tan et al., 2019). RNase readily degrades miRNA in the plasma; thus, researchers have used exosomes produced by lentiviral overexpression of miR-21 as a carrier to deliver miR-21 to the inner ear, preventing hearing loss from ischemia-reperfusion (Hao et al., 2022). Injecting miR-375 agomir can alleviate nasal mucosa inflammation in allergic rhinitis mice (Wang et al., 2018). However, the ability to overexpress FOXG1 and miRNA efficiently in the inner ear remains limited. Preventing and treating hair cell damage and hearing loss caused by cisplatin through gene therapy is a new focus of inner ear research.

**Figure 10:**
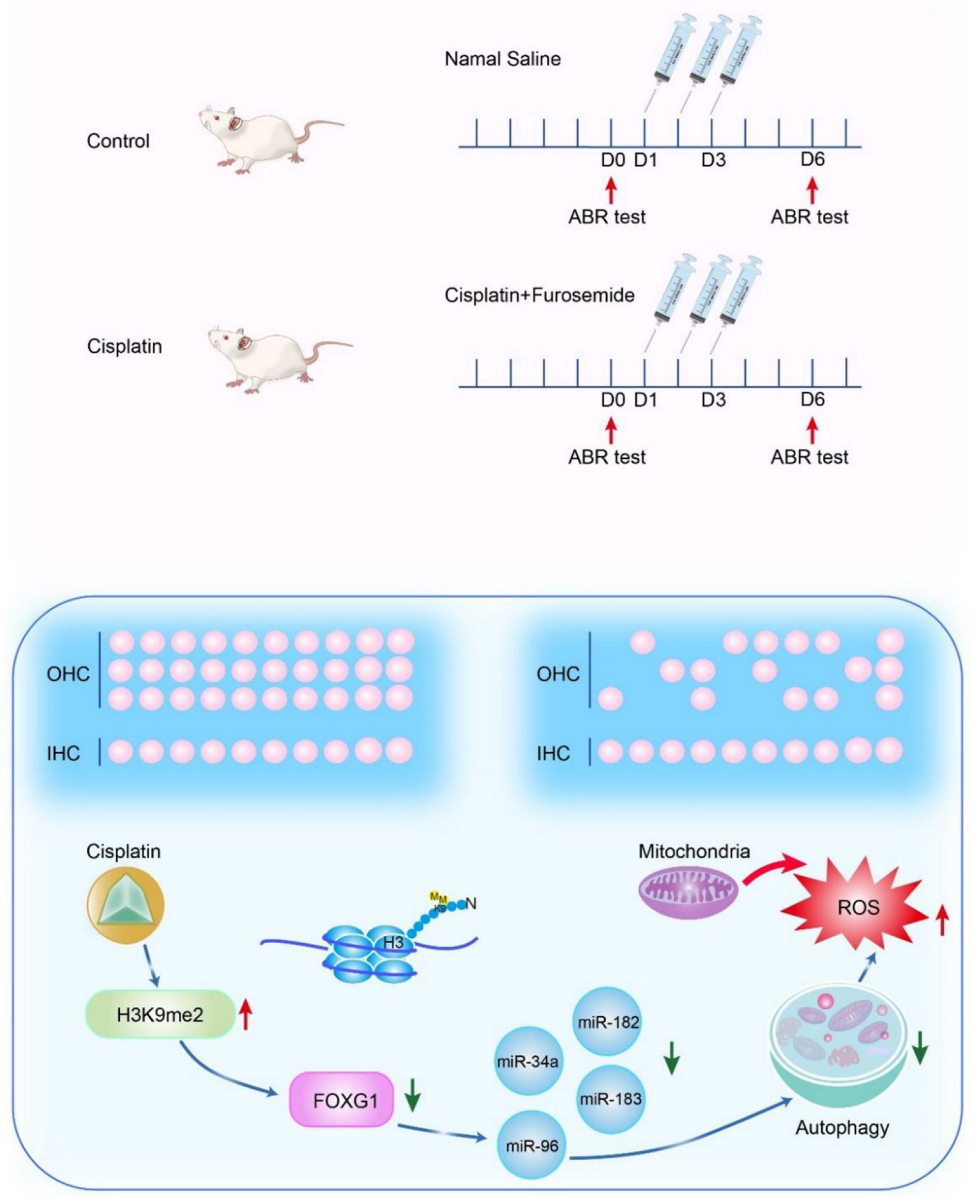
Mechanism of FOXG1-related epigenetic modifications in cisplatin-induced HC damage.

We used the cisplatin ototoxic OC-1 cell line and the CBA/CaJ mouse model to determine FoxG1's role and mechanism in cisplatin-induced ototoxic HC degeneration. We found that cisplatin decreased FOXG1 expression and autophagy levels, and that H3K9me2 played a role in cisplatin-induced ototoxicity. Reduced FOXG1 expression resulted in a series of miRNA changes that reduced autophagy activity and led to ROS accumulation and subsequent cochlear HC death. Following miRNA inhibition, autophagy levels decreased, but ROS levels and the apoptosis ratio increased, leading to HC death (Figure 10). By epigenetic regulation, we found that combining G9a inhibition with cisplatin has the potential to rescue hearing trauma and sensory hair cells loss. This protocol might represent an improvement for patients to limit chemotherapy-induced hearing loss. Our study has identified a potential target for future auditory HC protection against cisplatin injury.

## Materials and methods

### Animals

Six-week-old male SPF-grade CBA/CaJ mice (RRID: IMSR_JAX:000654) were obtained from SPF (Beijing) Biotechnology Co. They were kept for 1 week after purchase to allow them to adapt to the environment before the experiments began. All experimental work passed the ethical review for animal experiments and was conducted according to Committee on Animal Research policies at Tongji Medical College, Huazhong University of Science and Technology.

### Drugs

Cisplatin was obtained from Yunnan Botanical Pharmaceutical Co., furosemide from Henan Runhong Pharmaceutical Co., and BIX01294 from APExBIO (cat no. A1909).

### *In vivo* drug treatment

The *in vivo* experiments used male SPF-grade CBA/CaJ mice. The cisplatin group were given furosemide and cisplatin to create the animal model. First, furosemide at 200 mg/kg was injected intraperitoneally. Next, half an hour later, cisplatin at 0.5, 1, 1.5, or 2 mg/kg was given subcutaneously. Then, 1 h later, 0.5 ml isotonic sodium chloride solution was given intraperitoneally. The control group was injected with isotonic sodium chloride solution. All injections were performed for three consecutive days. In the cisplatin +BIX01294 group, BIX01294 at 20 mg/kg or 40 mg/kg was injected intraperitoneally. BIX01294 was injected on the day before the start of cisplatin injection and half an hour before each furosemide injection.

### ABR

The ABR was measured before and 3 days after treatment. After anesthesia induction, the mice were placed in a soundproof room and kept warm with a warm water bag. Electrodes were inserted into the ear to be tested, the contralateral ear, and subcutaneously in the middle of the head. A TDT device measured the click ABR and tone burst ABR at 8, 16, 24, 32, and 40 kHz. Each frequency was measured from 90 dB and lowered by 10 dB each time until there was no response to determine the threshold for each frequency.

### Cell culture

The OC-1 cells were cultured in a 37°C incubator with 5% CO_2_ in a complete culture medium of high-glucose DMEM (Hyclone) supplemented with 10% fetal bovine serum (Gibco) and 50 units/ml penicillin. Cultured cells were passaged when they reached 80%−90% confluence. Cells were digested with 0.25% trypsin, and the digestion was terminated with the complete medium. The cells were collected in a 5 ml EP tube, centrifuged at 1,500 rpm for 5 min at room temperature, and the supernatant was discarded. Thereafter, 2 ml of the complete culture medium was added to resuspend and inoculate an appropriate amount in a 10 cm Petri dish.

### CCK-8

The cells were seeded in 96-well plates at the appropriate density and treated with the appropriate drugs 24 h after inoculation. Each group comprised six sub-wells. After treatment, the drugs were removed, and 100 μl DMEM containing 10% CCK-8 reagent was added to each well. Each well's absorbance at 450 nm was measured after 30 min and 1 h in the 37°C incubator.

### Transfection

siRNA-*Foxg1* was designed and synthesized by Tsingke Biotechnology Co. to knock down *Foxg1* expression in OC-1 cells, *Foxg1* overexpressing plasmid was designed and synthesized by Shanghai GenePharma Co. to upregulate *Foxg1* expression. The miRNA inhibitors and miRNA mimics were designed and synthesized by Guangzhou RiboBio Co. to inhibit or increase target miRNA expression. The OC-1 cells were passaged, seeded in six-well plates for 24 h, cultured to 50%−60% confluence, and transfected in Opti-MEM using lipo3000 reagent. At 6–8 h after transfection, the Opti-MEM was replaced with the complete culture medium.

### Rt-PCR

Total RNA was extracted from cells using TRIzol reagents. A miRNA rt-PCR reagent (Guangzhou RiboBio Co.) and a reverse transcription kit (Takara) to perform miRNA reverse transcription and rt-PCR.

### Protein extraction

Cells were digested using 0.25% trypsin, which was stopped using a complete culture medium. Briefly, cells were collected in a 1.5 ml EP tube, centrifuged at 1,500 rpm for 5 min at room temperature, and the supernatant was discarded. Next, cells were resuspended in a RIPA buffer containing phosphatase inhibitors, protease inhibitors, and PMSF and left to lyse on ice for 20 min. Then, a 5 × loading buffer was added to the lysed mixture, which was boiled at 95°C for 15 min and stored at −20°C.

The cochlea was dissected, removed, and soaked in phosphate-buffered saline (PBS). Next, a pre-chilled RIPA buffer containing phosphatase inhibitors, protease inhibitors, and PMSF was added. Then, the cochlea was crushed and sonicated at 20% energy for 5 s before centrifugation at 12,000 rpm and 4°C for 10 min in a low-temperature high-speed centrifuge. Finally, the supernatant was aspirated, and a 5 × loading buffer was added to the mixture, which was boiled at 95°C for 15 min and stored at −20°C.

### Western blotting

SDS-PAGE gel electrophoresis was performed to separate the proteins. The proteins on the gel were transferred to PVDF membranes, which were blocked with 5% nonfat milk in TBST for 1 h at room temperature on a shaker. The membrane was placed in the primary antibody, incubated overnight using a refrigerator shaker, washed three times with TBST for 5 min each, and then incubated with a 1:5,000 secondary antibody for 1 h at room temperature. Washing with TBST was performed thrice for 5 min each, and exposure to film was achieved using an ECL solution in a dark room. The films were developed, fixed, and air dried. After scanning the films, we analyzed the immunoblot bands using Image J. The primary antibodies used were anti-FOXG1 antibody (Abcam, ab18259), anti-LC3B antibody (Sigma-Aldrich, L7543), anti-G9a antibody (Abcam, ab185050), and anti-H3K9me2 antibody (Abcam, ab176882).

### Flow cytometry

Mito-SOX Red (Thermo Fisher Scientific) was used to analyze mitochondrial ROS production. After trypsinization, the OC-1 cells were collected via centrifugation and washed with PBS. The cell pellets were then resuspended in a solution containing Mito-SOX Red for 15 min at 37°C in the dark and analyzed via flow cytometry (FACSCalibur, BD Biosciences,).

FITC/annexin V (BD Biosciences) was used to analyze apoptosis and PI to differentiate between live and dead cells. The OC-1 cells were trypsinized and collected via centrifugation at 1,000 rpm for 5 min, washed with PBS, resuspended in binding buffer, and aliquoted at 1 × 10^5^ cells (100 μl) into a 5 ml flow tube. FITC/annexin V and PI were added to the tube, and the mixture was vortexed gently, incubated at room temperature for 15 min in the dark, and analyzed via flow cytometry within 1 h.

### TEM

The OC-1 cells were fixed in 2.5% glutaraldehyde (Sigma-Aldrich) for 24 h and 1% osmic acid (Sigma-Aldrich) for 1–2 h, dehydrated with acetone (Sinopharm Chemical Reagent), and embedded with Araldite CY212 (TAAB). Ultrathin sections were stained with alcohol uranyl acetate (Polysciences) and alkaline lead citrate (Sigma-Aldrich). The sections were gently washed with distilled water and observed under a JEM 1230 transmission electron microscope (JEOL Ltd.).

### Immunofluorescence staining

Anti-FOXG1 antibody (Abcam, ab18259), anti-LC3B antibody (Sigma-Aldrich, L7543), anti-H3K9me2 antibody (Abcam, ab176882), Moysin7a (Proteus Biosciences, 25-6790), rhodamine phalloidin (Yeasen), and DAPI (Solarbio) were used to analyze the FOXG1 expression and detect autophagy, H3K9me2, HCs, and microfilament structure and nucleus, respectively.

The samples were incubated in 4% paraformaldehyde (Sigma-Aldrich) for 1 h and then blocked with 0.5% Triton X-100 (blocking medium) for 1 h. The primary antibodies were then added at a 1:400–1:1,000 dilution and incubated overnight at 4°C. The samples were washed thrice with PBST, incubated with fluorescent secondary antibodies for 1 h at room temperature in the dark, rewashed thrice with PBST, and reincubated with rhodamine phalloidin and DAPI for 30 min in the dark. After sealing the slides with clear nail polish, we imaged them using a confocal microscope.

### Data analysis

All data were presented as means ± SDs. All experiments were repeated at least thrice. Statistical analysis was performed using Microsoft Excel and GraphPad Prism 8. Statistical significance was determined using a two-tailed unpaired *t*-test when comparing two groups and using one-way ANOVA and Dunnett's multiple comparison test when comparing more than two groups. *p*-values of <0.05 were considered statistically significant.

## Data availability statement

The original contributions presented in the study are included in the article/Supplementary material, further inquiries can be directed to the corresponding author/s. The raw data from the figures presented in the study are publicly available. This data can be found here: https://www.jianguoyun.com/p/DSZBQ_EQmd-ECxjv094EIAA.

## Ethics statement

The animal study was reviewed and approved by the Committee on Animal Research of Tongji Medical College, Huazhong University of Science and Technology.

## Author contributions

W-jK and Z-hH conceived and designed the study and reviewed and edited the manuscript. Y-rM, S-yZ, ML, Y-yD, and XH performed the experiments. Z-hH, Y-rM, and S-yZ analyzed the data and wrote the manuscript. All authors have read and approved the published version of the manuscript.

## Supplementary material

Supplementary Figure 1 Cell viability and apoptosis flow cytometry results for the OC-1 cells treated with different BIX01294 concentrations. **(A)** CCK-8 cell viability after treatment with different BIX01294 concentrations for 24 h. **(B)** Annexin V flow cytometry of apoptosis in cells treated with different BIX01294 concentrations for 24 h. **(C)** Quantification of the apoptosis ratio in **(B)**. **(D)** Quantification of the cell death ratio in B. ^*^*p* < 0.05, ^**^*p* < 0.01, ^***^*p* < 0.001.

Supplementary Figure 2 Mito-SOX flow cytometry of OC-1 cells treated with BIX01294. **(A)** ROS level of treated with different BIX01294 concentrations for 24 h. **(B)** Quantification of the data in A. ^*^*p* < 0.05.
