# Supplementary material for: Role and mechanism of FOXG1-related epigenetic modifications in cisplatin-induced hair cell damage
Source: Front Mol Neurosci. 2023 Apr 26;16:1064579. doi: 10.3389/fnmol.2023.1064579 (PMC10169754; doi:10.3389/fnmol.2023.1064579)
Supplement: Supplementary Figure 1 — Cell viability and apoptosis flow cytometry results for the OC-1 cells treated with different BIX01294 concentrations. (A) CCK-8 cell viability after treatment with different BIX01294 concentrations for 24 h. (B) Annexin V flow cytometry of apoptosis in cells treated with different BIX01294 concentrations for 24 h. (C) Quantification of the apoptosis ratio in (B). (D) Quantification of the cell death ratio in B. *p < 0.05, **p < 0.01, ***p < 0.001. [file Data_Sheet_1.pdf]

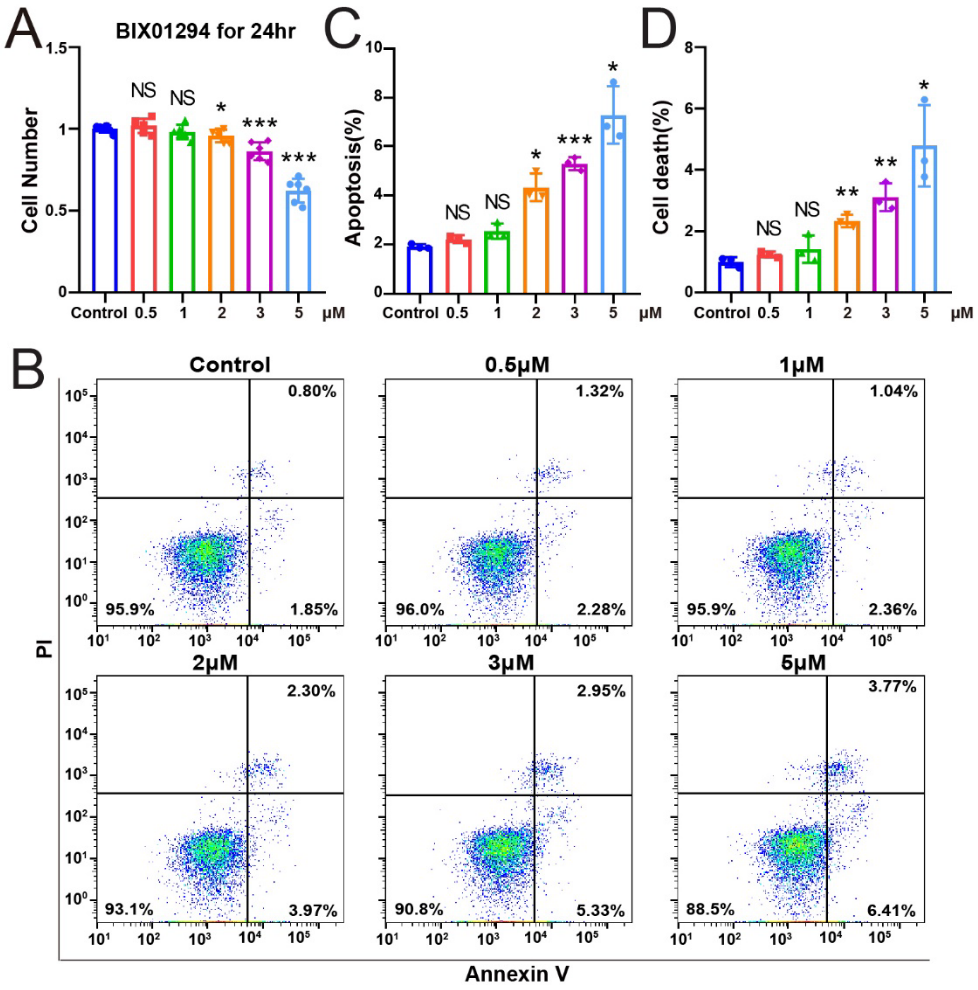

Supplementary Figure 1. Cell viability and apoptosis flow cytometry results for the OC-1 cells treated with different BIX01294 concentrations. (A) CCK-8 cell viability after treatment with different BIX01294 concentrations for 24 h. (B) Annexin V flow cytometry of apoptosis in cells treated with different BIX01294 concentrations for 24 h. (C) Quantification of the apoptosis ratio in B. (D) Quantification of the cell death ratio in B. $^{*}p < 0.05$, $^{**}p < 0.01$, $^{***}p < 0.001$

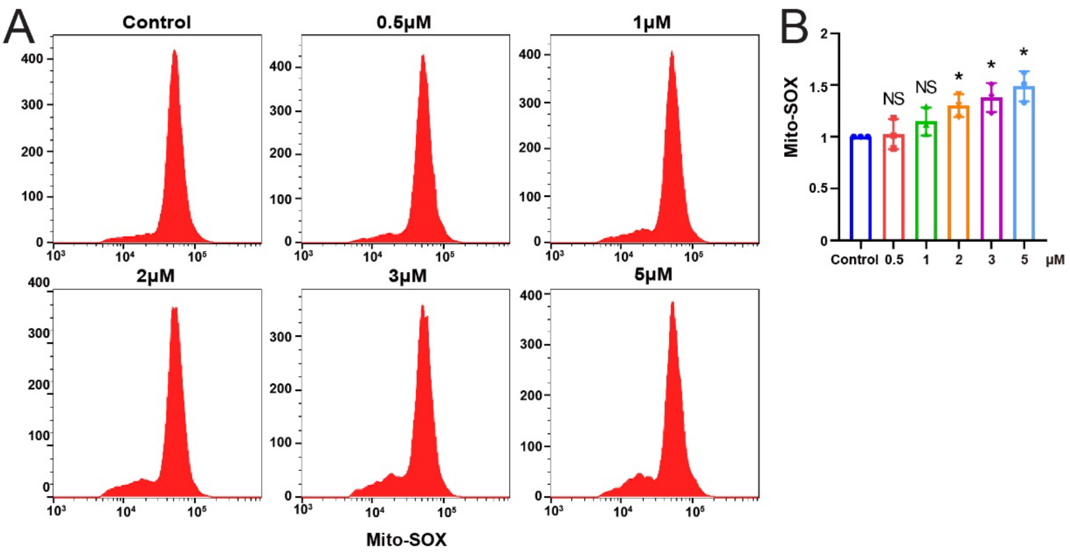

Supplementary Figure 2. Mito-SOX flow cytometry of OC-1 cells treated with BIX01294. (A) ROS level of treated with different BIX01294 concentrations for 24 h. (B) Quantification of the of the data in A. $^{*}p$ <0.05
